# Swallowing Exercise Evaluated Using High-Density Surface Electromyography in Patients with Head and Neck Cancer: Supplementary Analysis of an Exploratory Phase II Trial

**DOI:** 10.3390/medicina59122120

**Published:** 2023-12-04

**Authors:** Kohei Yoshikawa, Takao Hamamoto, Yuki Sato, Kohei Yumii, Nobuyuki Chikuie, Takayuki Taruya, Takashi Ishino, Yuichiro Horibe, Kota Takemoto, Manabu Nishida, Tomohiro Kawasumi, Tsutomu Ueda, Yuichi Nishikawa, Yukio Mikami, Sachio Takeno

**Affiliations:** 1 Department of Clinical Support, Division of Rehabilitation, Hiroshima University Hospital, 1-2-3, Kasumi, Minami-ku, Hiroshima 734-8551, Japan; kohei@hiroshima-u.ac.jp (K.Y.); mikamiy@hiroshima-u.ac.jp (Y.M.); 2 Department of Otorhinolaryngology, Head and Neck Surgery, Hiroshima University Hospital, 1-2-3, Kasumi, Minami-ku, Hiroshima 734-8551, Japan; sato0123@hiroshima-u.ac.jp (Y.S.); yumiik@hiroshima-u.ac.jp (K.Y.); housejak@hiroshima-u.ac.jp (N.C.); ttaruya@hiroshima-u.ac.jp (T.T.); tishino@hiroshima-u.ac.jp (T.I.); horibey@hiroshima-u.ac.jp (Y.H.); kota61@hiroshima-u.ac.jp (K.T.); nm1027@hiroshima-u.ac.jp (M.N.); kwtm2022@hiroshima-u.ac.jp (T.K.); uedatsu@hiroshima-u.ac.jp (T.U.); takeno@hiroshima-u.ac.jp (S.T.); 3 Institute of Science and Engineering, Faculty of Frontier Engineering, Kanazawa University, Kanazawa 920-1192, Japan; yuichi@se.kanazawa-u.ac.jp

**Keywords:** head and neck cancer, electromyogram, chemoradiotherapy, swallowing function, dysphagia, rehabilitation

## Abstract

*Background and Objectives:* Muscle strength evaluation using high-density surface electromyography (HD-sEMG) was recently developed for the detailed analysis of the motor unit (MU). Detection of the spatial distribution of sEMG can detect changes in MU recruitment patterns resulting from muscle-strengthening exercises. We conducted a prospective study in 2022 to evaluate the safety and feasibility of transcutaneous electrical sensory stimulation (TESS) therapy using an interferential current device (IFCD) in patients with head and neck squamous cell carcinoma (HNSCC) undergoing chemoradiotherapy (CRT), and reported the safety and feasibility of TESS. We evaluated the efficacy of swallowing exercises in patients with HNSCC undergoing CRT and determined the significance of sEMG in evaluating swallowing function. *Materials and Methods:* In this supplementary study, the patients performed muscle-strengthening exercises five days a week. The association of the effects of the exercises with body mass index, skeletal muscle mass index, HD-sEMG, tongue muscle strength, and tongue pressure were evaluated. *Results:* We found significant correlations between the rate of weight loss and skeletal muscle mass index reduction and the rate of change in the recruitment of the MU of the suprahyoid muscle group measured using HD-sEMG. *Conclusions:* We believe that nutritional supplementation is necessary in addition to muscle strengthening during CRT.

## 1. Introduction

Chemoradiotherapy (CRT) is currently the standard treatment for locally advanced head and neck cancer [1,2]. However, CRT causes mucositis, dermatitis, muscle and nerve dysfunction, and tissue fibrosis, resulting in post-treatment dysphagia [3]. Among the associated adverse events associated with CRT, dysphagia is a major detriment to quality of life. Several recent studies have reported the relationship between nutritional status and swallowing function; however, patients with head and neck cancer are prone to undernutrition because of the impaired food passage associated with the primary tumor, swallowing pain associated with radiation-induced mucositis, anorexia, and nausea associated with chemotherapy during treatment. Therefore, many studies have reported the importance of nutritional management and early intervention of swallowing rehabilitation in preventing dysphagia [4,5,6].

Swallowing exercises are often used in rehabilitation during CRT to increase muscle strength and prevent muscle atrophy. In general, muscle training is a well-established intervention for muscle strengthening, as it is believed to produce muscle hypertrophy effects and changes in the firing threshold and discharge rate of motor units (MUs), in addition to increasing the muscle output power [7,8]. Muscle strength evaluation using high-density surface electromyography (HD-sEMG) can provide detailed information about MUs. Changes in MU recruitment patterns as a result of muscle-strengthening exercises can be detected via analysis of the sEMG spatial distribution [9,10,11,12].

**Figure 1:**
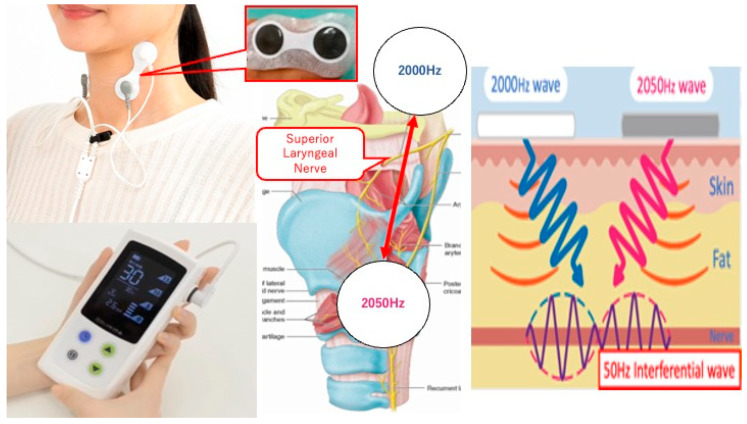
Interferential current device named “Gentle-Stim”.

In 2022, we conducted a prospective study to evaluate the safety and feasibility of transcutaneous electrical sensory stimulation (TESS) therapy using an interferential current device (IFCD) named “Gentle-Stim” (Food Care Co., Ltd. Kanagawa, Japan, medical device certification number: 227AHBZX00026000) in patients with head and neck squamous cell carcinoma (HNSCC) undergoing CRT (Figure 1) [13]. The primary endpoint was the feasibility of TESS for such patients; it was concluded that TESS was feasible until the end of treatment. To establish further studies examining the efficacy of TESS in patients with head and neck cancer receiving CRT, we included physical assessment, tongue pressure, and HD-sEMG of the suprahyoid muscle group (SHMG) as secondary endpoints because in order to study the effectiveness of TEES, swallowing function must be measured in some way, and these measurement values must be specifically quantified.

Few studies have investigated changes in the recruitment patterns of swallowing MUs in the SHMG during CRT. Therefore, the present study evaluated the effects of swallowing exercises in patients with HNSCC undergoing CRT. We also aimed to determine the significance of sEMG in evaluating swallowing function. Here, we present our results and discuss the existing literature.

Gentle-Stim is an inferential current device manufactured by Food Care Co., Ltd. (Kanagawa, Japan). Electrodes placed across the hyoid bone and thyroid cartilage create interference waves, stimulate the superior laryngeal nerve, and improve laryngeal sensation.

## 2. Materials and Methods

### 2.1. Ethics

The study protocol was approved on 17 March 2022 by the Certified Clinical Research Committee of Hiroshima University (certification number: CRB210005), registered with the Japan Registry of Clinical Trials (jRCTs062220008), and submitted to the Ministry of Health, Labour and Welfare. Written informed consent was obtained from each participant, and the study was conducted in accordance with the tenets of the Declaration of Helsinki.

### 2.2. Study Objectives and Eligibility Criteria

This single-center, exploratory, single-arm prospective study was conducted to evaluate the safety of TESS in patients enrolled and treated between 13 April 2022 and 30 March 2023. Ten patients with locally advanced head and neck cancer who underwent CRT were selected from Hiroshima University Hospital. The eligibility criteria were (1) patients who underwent CRT for head and neck cancer at Hiroshima University Hospital; (2) patients who received 70 Gy of radiation to the laryngopharyngeal area, including the nasopharynx, oropharynx, hypopharynx, or larynx; (3) patients >20 years old at the time of consent; and (4) patients who provided written consent for participation in this study. The exclusion criteria were (1) patients with a history of radiation therapy administration in the head and neck region; (2) patients with a history of tracheostomy; (3) patients with a history of radiation therapy mainly in an area other than the laryngopharyngeal area; (4) patients with pacemakers and implantable cardioverter-defibrillators; (5) patients with difficulty wearing an IFCD on the neck; (6) patients with many inconveniences in daily life (performance status 2 or higher); (7) pregnant and breastfeeding patients and those of reproductive age; and (8) patients who were judged to be inappropriate by the principal investigator or the research coordinator. This study was designed to evaluate the safety and feasibility of TESS using IFCD in patients with HNSCC undergoing CRT. The details of the 10 eligible cases were previously reported [13]. A supplementary objective was to assess the efficacy of HD-sEMG in swallowing exercises.

### 2.3. Measurement Items

#### 2.3.1. Physical Status

Patients’ height and weight were measured, and their body mass index (BMI) was calculated. Their skeletal muscle mass was calculated using bioelectrical impedance analysis with the InBody S10 (InBody Japan Co., Ltd., Tokyo, Japan), and their skeletal muscle mass index (SMI) was calculated [14].

#### 2.3.2. HD-sEMG

**Figure 2:**
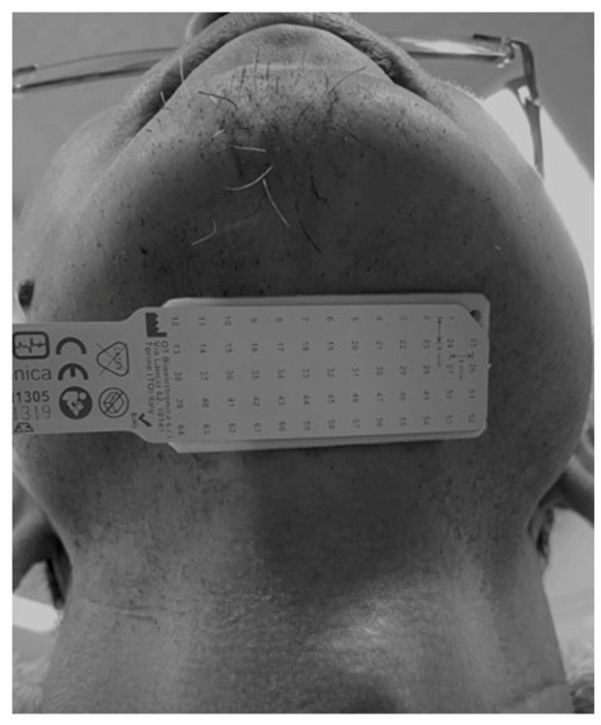
High-density surface electromyography (HD-sEMG).

The muscle activity of the SHMG was measured using HD-sEMG. The electrodes were 64 channels in 13 rows and 5 columns of a 1 mm diameter sheet (GR04MM1305, OT Bioelettronica Co. Ltd., Torino, Italy). The electrode sheet was affixed to the anterior cervical midline between the mandible and hyoid bone (Figure 2). To detect stable muscle activity, 50 bipolar surface electromyographic (sEMG) signals were derived from 55 electrodes, with the exclusion of the electrodes at both ends. Signals from each electrode were captured using an 18-bit A/D converter and a bandpass filter of 10–500 Hz (EMG-USB2+, OT Bioelettronica). The EMG signals were analyzed using analysis software (MATLAB 2019a, MathWorks, Inc., Natick, MA, USA), and the root mean square (RMS) of the amplitude was calculated as the index of muscle activity. The RMS value was calculated as the average of values derived from the 50 bipolar sEMG signals. Furthermore, the RMS values were normalized with respect to the values obtained at 0% of MVC. Also, the changes in the spatial distribution pattern of muscle activity within the electrode were determined using the coefficient of variation (CoV), (SD/Ave × 100, CoV force) relative to the RMS value.

The electrode sheet was affixed to the anterior cervical midline between the mandible and hyoid bone. Signals from each electrode were captured and the EMG signals were analyzed using analysis software (MATLAB 2019a, MathWorks, Inc., MA, USA).

#### 2.3.3. Measurements of Tongue Muscle Strength

Tongue muscle strength was measured using a tongue strength meter (Takei Scientific Instruments Co. Ltd., Niigata, Japan). The maximum tongue muscle strength produced by pushing the tongue depressor was measured using isometric contraction for 3 s. The highest value was considered the maximum tongue strength. A ramp-up task for progressive muscle power exertion was performed against the maximum voluntary contraction (MVC) obtained during tongue raising. The examinee progressively increased the muscle strength at 10% MVC/s from 0% to 80% MVC while receiving visual feedback of the tongue muscle strength data displayed on the monitor. The HD-sEMG was recorded during this task in accordance with the ramp-up task [15]. Among the EMG signals from 0% to 80% MVC, the EMG data at three points (50%, 60%, and 65% MVC) were analyzed.

#### 2.3.4. Tongue Pressure

Tongue pressure was measured using the JMS tongue pressure monitor TPM-01 (JMS Co., Ltd., Hiroshima, Japan). A tongue pressure probe was inserted into the oral cavity, and the base of the probe was lightly cupped by the upper and lower incisors to fix the mandible. The probe was fixed between the anterior part of the tongue and hard palate, and the patient’s tongue was pressed against the probe as hard as possible (maximum tongue pressure value). The maximum tongue pressure (MTP) was measured twice, and the larger value was used as the maximum value.

### 2.4. Swallowing Muscle Exercise

Swallowing exercises were performed five days a week for eight weeks, with mouth-opening training, cervical isometric contraction exercises, and Shaker exercises. For the mouth-opening training, three sets of 20 opening movements were performed at the maximum opening position. For the cervical isometric contraction exercise comprised 15 sets of 5 s contractions were performed. In the Shaker exercise, one set of 60 s of continuous head raising and 30 repetitions of head raising were performed.

### 2.5. Statistical Analysis

Patients’ height, weight, BMI, SMI, and tongue pressure were measured before and after treatment. Two parameters, the CoV and RMS, were calculated using the HD-sEMG. The Wilcoxon signed-rank test was used to compare the results before and after each treatment. The rate of change (measured value before start–measured value after treat-ment/measured value before start × 100) was calculated. The CoV and RMS were measured before and after treatment, and the rate of change before and after treatment was calculated. Correlation coefficients were calculated for each of the three physical assessment items and the two parameters of the HD-sEMG. Statistical analyses were performed using JMP Pro ver.16.2.0 (SAS Institute Inc., Cary, NC, USA). The threshold for statistical significance was set at *p* < 0.05 for each parameter.

## 3. Results

The 10 enrolled patients were all males. The clinical data of the 10 patients are presented in Table 1. The median patient age was 67 (45–76) years, and the ECOG PS performance status score was 0. The primary sites were the nasopharynx (one patient), hypopharynx (seven patients), and larynx (one patient), with one remaining unknown (one patient). Two, four, and four patients had clinical stages II, IVa, and IVb, respectively. For the treatment details shown in Table 2, six cases received induction chemotherapy (docetaxel, cisplatin, 5-FU) before CRT and four cases received two cycles of tri-weekly cisplatin, five cases received three cycles of tri-weekly cisplatin, and one case received seven cycles of weekly cetuximab during irradiation. As for the irradiation dose of radiation, nine patients received a prescribed dose of 70 Gy/35 fractions and one patient received 66 Gy/33 fractions; the patient was irradiated with a large field for an unknown primary cancer, resulting in severe mucositis, and RT was terminated based on clinical judgment. All patients were treated using intensity-modulated radiation therapy (IMRT) as the radiation technique. Combination chemotherapy consisting of cisplatin was administered to all patients, except one who received cetuximab owing to decreased renal function.

The values of the CoV and RMS correlation coefficients between the rate of change in body weight and HD-sEMG parameters were, respectively, CoV *p* = 0.28, r = 0.37 and RMS *p* = 0.009, r = 0.77 at 50% MVC, CoV *p* = 0.05, r = 0.63 and RMS *p* = 0.005, r = 0.80 at 60% MVC, and CoV *p* = 0.05, r = 0.63 and RMS *p* = 0.005, r = 0.80 at 65% MVC. The values of the CoV and RMS correlation coefficients between the rate of change in SMI and HD-sEMG parameters were, respectively, CoV *p* = 0.21, r = 0.43 and RMS *p* = 0.04, r = 0.65 at 50% MVC, CoV *p* = 0.08, r = 0.57 and RMS *p* = 0.01, r = 0.76 at 60% MVC, CoV *p* = 0.012, r = 0.74 and RMS *p* = 0.023, r = 0.70 at 65% MVC. The values of the CoV and RMS correlation coefficients between the rate of change in tongue pressure and HD-sEMG parameters were, respectively, CoV *p* = 0.97, r = −0.013 and RMS *p* = 0.97, r = −0.01 at 50% MVC, CoV *p* = 0.73, r = 0.12 and RMS *p* = 0.73, r = 0.12 at 60% MVC, and CoV *p* = 0.93, r = 0.03 and RMS *p* = 0.93, r = 0.03 at 65% MVC.

In HD-sEMG, a significant correlation was observed between the rate of change in body weight and the rate of change in the CoV and RMS. Additionally, a significant correlation was also found between the rate of change in SMI and the rate of change in the CoV and RMS. However, no significant correlation was observed between the rate of change in tongue pressure and the rate of change in the CoV and RMS. On the other hand, there were no significant differences in BMI, SMI, tongue pressure, the CoV, or the RMS before and after treatment (Table 3).

## 4. Discussion

Although CRT for head and neck cancer has been reported to cause tissue scarring, muscle atrophy, and muscle weakness [3,16], there are no detailed reports on the effects of CRT on the MU of swallowing muscles. In the present study, the SHMG continuously performed swallowing exercises using TESS during CRT; we evaluated the changes in the MU of the swallowing muscle using HD-sEMG and obtained several findings.

Muscle strength is regulated by the muscle fibers and the nerve factors that activate them. Nerve factors control the recruitment and discharge rate of the MU during muscle contractions; the higher the number of MUs and discharge rate, the higher the muscle strength [9,10,11,12]. HD-sEMG is a method to spatially evaluate the MU firing behavior and can detect the MU recruitment pattern distribution and changes. The RMS reflects the muscle activation quantification of the MU, whereas the CoV indicates the spatial distribution of the muscle activity [10,17,18]. Muscle-strengthening exercises are thought to increase muscle strength by stimulating hypertrophy of the muscle fibers, lowering the recruitment threshold, and increasing the discharge rate [7,8], which has been proven in actual clinical practice [18].

In the present study, patients underwent eight weeks of muscle-strengthening exercises for the SHMG with TESS. Therefore, it was assumed that muscle exercises would increase the RMS and CoV before and after treatment; however, no such increase was observed. This may be because patients could not perform sufficient strength training due to the pain, fatigue, and nausea caused by mucositis, dermatitis, and stomatitis during CRT. Most of the training performed in this study involved weight-bearing and required physical exertion. Therefore, it seems likely that the patients undergoing CRT could not perform aggressive high-intensity exercises. The second factor is a decline in nutritional status. A high correlation was found between body weight or SMI change rate, an indicator of nutritional status, and RMS or CoV change rate. Patients with decreased body weight and SMI had a decreased discharge rate and distribution of MUs, that is, decreased swallowing muscle strength. Recently, the concept of sarcopenia has been proposed, with many reports that low nutrition causes dysphagia [19,20,21]. Regarding the causal relationship between undernutrition and dysphagia, a vicious cycle has been postulated in which undernutrition causes a decrease in the mass of swallowing-related muscles, muscle at-rophy, and contractility of muscle fibers, resulting in dysphagia [22]. Therefore, the present study’s results support the finding that the number of MUs decreases in an undernourished state [23]. As patients with head and neck cancer are at high risk of secondary sarcopenia due to the malnutrition and hypercatabolism associated with dysphagia during CRT, exercise and nutritional interventions are needed during treatment [24,25].

Although it has been hypothesized that tongue-raising movements correlate with electromyograms of the SHMG [26], no correlation was found between the rate of change in tongue pressure and the rate of change in the MU of the SHMG in the present study. Because the irradiation sites of the patients were mainly located around the larynx, the irradiation dose to the oral cavity was limited. Tongue pressure is thought to be generated by the internal and external lingual muscles and the SHMG, and the limited effect of radiation on the lingual muscles suggests that tongue pressure was not involved in the changes in the MU of the SHMG. Additionally, we believe that TESS did not affect the strength of the SHMG, as the TESS targeted the sensory nerve. Although the safety of TESS in CRT for HNSCC has been demonstrated in our previous study, no significant changes were observed before and after treatment. However, significant correlations were observed between the rate of weight loss and SMI reduction and the rate of change in the recruitment of MU of the SHMG. The results suggest that it would be needed to observe changes over time in HD-sEMG in future studies to demonstrate its efficacy in dysphagia.

This study had some limitations. First, the sample size was not enough to reach a definitive conclusion and this study was only a pre- and post-treatment comparison, and it is not clear at what stage during treatment each of the parameters changed. Therefore, whether the effects of muscle-strengthening exercises were limited to the first few weeks of treatment was not adequately measured. We realize the need to investigate the changes over time from a prophylactic perspective to reduce adverse events. As most head and neck cancer patients are male in general, all patients were male in this study. It should be mentioned that the gender difference in muscle mass could be relevant to HD-sEMG, muscle strength, and tongue pressure. Although we focused on the body weight, SMI, and MU of the SHMG, there were many acute adverse events during CRT, including mucositis, dermatitis, and vomiting. It was not clear which of these adverse events strongly influenced the treatment completion rate, oral intake maintenance rate, or length of hospital stay; therefore, continued data collection is needed.

## 5. Conclusions

Muscle-strengthening exercises were performed in patients undergoing CRT for head and neck cancer, and their effects before and after treatment were investigated using HD-sEMG. Although no significant changes were observed before and after treatment, significant correlations were observed between the rate of weight loss and SMI reduction and the rate of change in the recruitment of MU of the SHMG. Therefore, we believe that nutritional supplementation is necessary in addition to muscle strengthening during CRT. A detailed investigation of the changes over time may help us understand the effects of muscle strengthening and adverse events in detail.

## Figures and Tables

**Table 1:** Characteristics of the patients enrolled.

Characteristic	No. of Patients (*n* = 10)	(%)
Sex		
Male	10	100
Female	0	0
Age, years		
Median	67	
Range	45–76	
ECOG PS		
0	10	100
1	0	0
Primary sites of cancer		
Nasopharyngeal	1	10
Hypopharyngeal	7	70
Laryngeal	1	10
Unknown primary	1	10
TNM stage		
II	2	20
ⅣA	4	40
ⅣB	4	40
Diabetes		
+	1	10
−	9	90
Radiation therapy		
70 Gy/35 fractions	9	90
66 Gy/33 fractions	1	10
Concurrent Chemotherapy		
Cisplatin	9	90
Cetuximab	1	10

ECOG PS, Eastern Cooperative Oncology Group Performance Status.

**Table 2:** Details of patients background and treatment.

No.	Age/Sex	Primary Site	TNM/Stage	Treatment	Chemo Agent/Cycles
1	70/male	Hypopharynx	T1N2bM0/Stage IVA	CRT after IC 2cycles	Cisplatin/3 cycles
2	59/male	Nasopharynx	T1N1M0/Stage II	CRT after IC 2cycles	Cisplatin/2 cycles
3	73/male	Primary Unknown	T0N3bM0/Stage IVB	CRT	Cisplatin/3 cycles
4	76/male	Hypopharynx	T4aN2bM0/Stage IVA	BRT	Cetuximab/7 cycles
5	72/male	Hypopharynx	T2N3bM0/Stage IVB	CRT after IC 2cycles	Cisplatin/3 cycles
6	64/male	Hypopharynx	T4aN2cM0/Stage IVA	CRT after IC 2cycles	Cisplatin/2 cycles
7	45/male	Hypopharynx	T4bN3bM0/Stage IVB	CRT after IC 2cycles	Cisplatin/2 cycles
8	74/male	Hypopharynx	T4aN3bM0/Stage IVB	CRT after IC 2cycles	Cisplatin/2 cycles
9	51/male	Larynx	T2N0M0/Stage II	CRT	Cisplatin/3 cycles
10	64/male	Hypopharynx	T1N2bM0/Stage IVA	CRT	Cisplatin/3 cycles

CRT: chemoradiotherapy, IC: induction chemotherapy, BRT: bio-radiotherapy.

**Table 3:** Comparison of each indicator pre- and post-treatment.

Indicator	Pre-Treatment	Post-Treatment	*p*-Value
BMI, kg/m^2^	22.06 ± 3.61	20.99 ± 3.14	0.486
SMI, kg/m^2^	7.67 ± 1.07	7.24 ± 1.15	0.399
MTP, kpa	33.39 ± 4.87	30.80 ± 5.90	0.298
Coefficient of variation (50%), median [IQR]	6.47 (3.95–13.82)	5.89 (3.57–6.71)	0.405
Coefficient of variation (60%), median [IQR]	8.95 (5.39–16.26)	6.31(3.67–7.35)	0.256
Coefficient of variation (65%), median [IQR]	7.42 (3.72–11.33)	6.71 (5.51–7.93)	0.939
Root mean square (50%), median [IQR]	1.14 (1.06–1.25)	1.17 (0.95–1.24)	0.496
Root mean square (60%), median [IQR]	1.24 (1.12–1.31)	1.26 (0.95–1.39)	0.570
Root mean square (65%), median [IQR]	1.26 [1.18–1.33]	1.35 [1.02–1.42]	0.969

BMI, the body mass index. SMI, the skeletal muscle mass index. MTP, maximum tongue pressure. IQR, interquartile range. Data are presented as the mean ± standard deviation, median (25%–75% IQR), or number of patients (%).

## Data Availability

The data presented in this study are available upon reasonable request from the corresponding authors.
